# Biodiversity Effects on Plant Stoichiometry

**DOI:** 10.1371/journal.pone.0058179

**Published:** 2013-03-04

**Authors:** Maike Abbas, Anne Ebeling, Yvonne Oelmann, Robert Ptacnik, Christiane Roscher, Alexandra Weigelt, Wolfgang W. Weisser, Wolfgang Wilcke, Helmut Hillebrand

**Affiliations:** 1 Institute for Chemistry and Biology of the Marine Environment, Carl von Ossietzky University of Oldenburg, Wilhelmshaven, Germany; 2 Institute of Ecology, Friedrich-Schiller-University Jena, Jena, Germany; 3 Geoecology/Geography, Eberhard Karls-University Tübingen, Tübingen, Germany; 4 UFZ, Helmholtz Centre for Environmental Research, Department of Community Ecology, Halle, Germany; 5 Institute of Biology, University of Leipzig, Leipzig, Germany; 6 Terrestrial Ecology, Department of Ecology and Ecosystem Management, Center of Life and Food Sciences Weihenstephan; Technische Universität München, Freising, Germany; 7 Geographic Institute, University of Berne, Berne, Switzerland; University of Zurich, Switzerland

## Abstract

In the course of the biodiversity-ecosystem functioning debate, the issue of multifunctionality of species communities has recently become a major focus. Elemental stoichiometry is related to a variety of processes reflecting multiple plant responses to the biotic and abiotic environment. It can thus be expected that the diversity of a plant assemblage alters community level plant tissue chemistry. We explored elemental stoichiometry in aboveground plant tissue (ratios of carbon, nitrogen, phosphorus, and potassium) and its relationship to plant diversity in a 5-year study in a large grassland biodiversity experiment (Jena Experiment). Species richness and functional group richness affected community stoichiometry, especially by increasing C:P and N:P ratios. The primacy of either species or functional group richness effects depended on the sequence of testing these terms, indicating that both aspects of richness were congruent and complementary to expected strong effects of legume presence and grass presence on plant chemical composition. Legumes and grasses had antagonistic effects on C:N (−27.7% in the presence of legumes, +32.7% in the presence of grasses). In addition to diversity effects on mean ratios, higher species richness consistently decreased the variance of chemical composition for all elemental ratios. The diversity effects on plant stoichiometry has several non-exclusive explanations: The reduction in variance can reflect a statistical averaging effect of species with different chemical composition or a optimization of nutrient uptake at high diversity, leading to converging ratios at high diversity. The shifts in mean ratios potentially reflect higher allocation to stem tissue as plants grew taller at higher richness. By showing a first link between plant diversity and stoichiometry in a multiyear experiment, our results indicate that losing plant species from grassland ecosystems will lead to less reliable chemical composition of forage for herbivorous consumers and belowground litter input.

## Introduction

Recent years have seen the rise of a strong body of literature examining the effects of biodiversity on ecosystem functioning (BDEF), which has been triggered by increasing concerns about potential consequences of loosing species in ecosystems worldwide. Recent syntheses of BDEF research in experimental ecosystems concluded that the loss of biodiversity reduces ecosystem process rates and stability [1]–[3]. However, there is substantial concern that many previous studies underestimate the strength of BDEF relationships because biodiversity effects become stronger in more complex settings and many of the previous experiments were restricted to a low maximum diversity [4]. Most BDEF studies addressed single ecosystem processes (e.g. primary production), but the loss of biodiversity might impact single processes less than “ecosystem multifunctionality”, which is defined as the composite of multiple ecosystem processes [5]. Different species might contribute to different processes in ecosystems, and recent work has shown that the loss of species is more likely to influence multiple processes rather than single processes (e.g., [6]–[8]).

Ecological stoichiometry (ES) [9] ties multiple processes in ecosystems together as the relation between organisms demand for multiple elements and the availability of these elements in their resources has profound impact on process rates and the relative importance of different processes. The nutrient stoichiometry of plant tissue can be decisive for species interactions with other trophic levels (herbivory, pathogen infestation) and nutrient recycling [10], [11]. Consequently, the ratios of carbon:nutrients can be used as a main predictor of the relative role of herbivory and detritus pathways in ecosystems [12], [13]. Different plant species may significantly affect the usage of different elements, and processes connected to these elements such as carbon (C) - based total primary productivity or cycling of nitrogen (N) or phosphorus (P), because plants separately consume anorganic elemental resources. Therefore, plants can show trade-offs in resource uptake and storage efficiency for different elements, which leads to higher plasticity in their elemental composition compared to animals, which consume resource packages [9], [14].

Unifying ES and BDEF research potentially creates new insights in how communities process available nutrients depending on the number of species involved. However, BDEF research has largely ignored stoichiometric considerations of ecosystem processes, whereas the analysis of ecological stoichiometry has rarely involved biodiversity because ES research often focused upon single species per trophic group or large-scale analyses in certain vegetation types or biomes (e.g., [15], [16]). In a pioneering study using algal microcosms, phytoplankton diversity was shown not only to alter primary productivity and P use, but also C:P ratios [17]. Ptacnik et al. [18] suggested on a more general level that plant elemental composition should vary with plant diversity (hypothesis H1), with different outcome depending on how much plant diversity affects resource use efficiency or storage for different elements. If species are highly complementary in their C-acquisition (e.g. different strategies in light acquisition through morphological or physiological traits), but not for mineral nutrients, then C should increase more rapidly with richness than N in community-wide chemical composition and C:nutrient ratios should increase (H1a). In contrast, if plant species show complementarity mainly for the uptake of organic or mineral nutrients but not for C-fixation, then increasing plant diversity should decrease C:nutrient ratios (H1b). The null hypothesis to both is that stoichiometry of community-wide chemical composition is independent of diversity because acquisition of different elements is so strongly coupled that no stoichiometric change is observed or because complementarity in resource acquisition traits is lacking.

It is important to test these potential diversity effects over time as nutrient availability and light limitation might change during community development, leading to time-dependent effects of diversity on stoichiometry (hypothesis H2). In addition to shifting mean ratios, plant diversity will also reduce the variance in chemical composition (hypothesis H3). This can be due to multiple mechanisms underlying diversity effects on resource uptake: More species increase the chance for selection or complementarity effects maximizing nutrient incorporation for each element across the assemblage and thereby lowering the variability of elemental concentrations. Alternatively, the variance in chemical composition can decrease with richness by a statistical averaging effect [19], where more species mask the signature of the stoichiometry of single species.

Here, we tested these three hypotheses on community-wide chemical composition using a grassland biodiversity experiment comprising communities of different species richness (1, 2, 4, 8, 16, and 60) and functional group richness and composition (1 to 4; legumes, grasses, small herb, tall herbs), the Jena Experiment [20]. We analyzed the relationship between plant diversity (species richness, functional group richness and functional composition) and the stoichiometry of plant chemical composition (C, N, P and K) over the first five years of the experiment.

## Materials and Methods

### Study Site and Experimental Design

The study was conducted in the Jena Experiment, a large biodiversity experiment established in 2002. The experimental site is located on the floodplain of the river Saale in Jena (Thuringia, Germany, 50°55′ N, 11°35′ E, 130 m a.s.l.) [20]. The area around Jena has a mean annual temperature of 9.3°C and an average annual precipitation of 587 mm [21].

The experimental design is described in detail in Roscher et al. [20]. Briefly, the main experiment comprises 82 plots of 20 m×20 m size. The soil of the experimental site is an Eutric Fluvisol. Due to flooding dynamics, the soil texture ranges from sandy loam close to the river Saale to silty clay with increasing distance from the river. Species were randomly drawn from a pool of 60 perennial species characteristic for Central European semi-natural, species-rich mesophilic grassland communities (Molinio-Arrhenatheretea [22]). According to the results of a cluster analysis of a literature-based matrix of functional traits, plant species were divided into 4 functional groups (16 grasses, 12 small herbs, 20 tall herbs, and 12 legumes). The experimental design ensures that the presence/absence of each functional group is minimally confounded with species number. Plant communities were established with different levels of species richness increasing on a logarithmic scale (1, 2, 4, 8, 16 and 60). Each species-richness level was replicated with 16 plots with different species composition, only species mixtures with 16 and 60 species were replicated on 14 and 4 plots, respectively. In addition to the main experiment, each experimental species was sown in replicated monocultures resulting in 120 plots of 3.5 m×3.5 m size.

To account for the gradient in soil characteristics, a block design was used with blocks arranged parallel to the river Saale. The plots were mown twice a year, in June and September, and mown material was removed. Additionally, plots were weeded at the beginning of the growing season and after first mowing to maintain the sown species combinations. Weeding was done mostly by hand [23].

### Sampling

Aboveground biomass was harvested from 2003 to 2007 at estimated peak standing biomass in late May prior to mowing. Plants were clipped at 3 cm above ground level in four rectangles of 20×50 cm size. In May 2005 only three samples were harvested. Biomass on small monoculture plots was sampled with two replicates. Sample location was selected randomly for each harvest leaving out the outer 70 cm of the plot. After harvest, plant material was sorted into sown species, species which were not sown at a particular plot and detached dead material. Biomass was dried at 70°C for at least 48 hours [23].

Biomass samples of the entire plant community per plot were shredded and milled for chemical analyses. Thus, all stoichiometric analyses were done on the mixture of pooled plot biomass. N and C concentrations were determined by an Elemental Analyzer (EA, Vario EL III, Elementar, Germany). Plant material were digested with HNO_3_ at 200°C using a microwave system (MARS5Xpress, CEM, Germany) to analyze P photometrically after irradiation with UV and oxidation with K_2_S_2_O_8_ with a Continuous Flow Analyzer (AutoAnalyzer, Bran&Luebbe, Germany) and K using atomic absorption spectrometry (AAS 240 FS, Varian, Germany) [24].

### Statistical Analyses

Bivariate molar ratios of C, N, P and K in the plant community were analyzed across the gradient of plant diversity with different levels of species and functional group richness. We present molar ratios as these are standard in ecological stoichiometry [9]. Since the six different molar ratios (C:N, C:P, C:K, N:P, N:K, P:K) were not independent of each other, a multivariate analysis of variance (MANOVA) was performed with the following factors: block, sown species richness, functional group richness, legume presence, grass presence. Legumes and grasses were explicitly tested because of potential strong impact on N (N_2_-fixing) and C (C-storage) concentration. We used the Pillai’s trace statistic, which is recommended to test for significant effects on interdependent response variables [25]. In cases of significant effects we used additional univariate tests for each ratio to analyze which ratios responded significantly to the factor. We opted for testing years separately in order to avoid a repeated measurement MANOVA which would be difficult to interpret.

We made two sensitivity analyses to test the robustness of our results. First, the MANOVA was repeated without the 60 species mixture to test for effects of this less replicated treatment. The results were comparable to those including the 60 species mixtures (Table S1), and changes were restricted to a reduced significance level reflecting the smaller statistical power (6 out of 25 results) and changes in the bivariate ratios becoming significant in the univariate tests of factors significant in the MANOVA (8 out of 25 results). Second, we changed the order of effects and tested functional group richness before testing species richness (see Table S2). Here we found that functional group richness often replaced species richness in importance, reflecting that changes in stoichiometry depended on functional and species richness in a comparable way. We present this alternative model in the supporting online material for comparison.

To analyze how increasing diversity levels effect the bivariate ratios and their variance, we used the software package generalized additive model for location, scale and shape (GAMLSS [26]). We preferred GAMLSS models over ordinary least square regression since they allow for fitting trends in mean and variance simultaneously. Models were fitted assuming normal distribution in the dependent variable. For both, mean and variance, two possible responses (none or linear) result in four possible combinations. The null model assuming no effect on either mean or variance was rejected in every case, such that we present the following models for comparison: Trend in mean, but no trend in variance (model m1), trend in mean and variance (m2) and no trend in mean but trend in variance (m4). The best fitting model per year and ratio was selected by AIC (Akaike’s Information Criterion) [27].

The results from MANOVA and GAMLSS were not always congruent for the different bivariate ratios. Generally, the MANOVA detects more significant diversity effects than the GAMLSS, which reflects the fact that the latter tested the direct association between richness and stoichiometry only, whereas the MANOVA extracts additional variation based on block and functional group presence and richness. Thus, in interpreting these outcomes, the MANOVA is the more powerful test for diversity effects on mean ratios, whereas the GAMLSS is the more powerful test for simultaneous changes in the variance.

Additionally, we tested how species diversity affects multi-element stoichiometry and the predictability of chemical composition. For each year, we used a Principal Components Analysis (PCA) on the concentrations of C, N, P, and K, producing two orthogonal axes explaining between 73% and 80% of the total variance in the different years. The first principal component was loaded by C concentration and opposed by P and K concentrations, whereas the second principal component was loaded by N concentrations alone. Factor loadings for PCA analysis (Table S3) were calculated with Statistica 8.0 (Statsoft, Tulsa Oklahoma). We used the origin of the PCA (0;0) as average stoichiometric composition and calculated the stoichiometric distance (*SDist*) for each of the 82 plots in each year. *SDist* is a multivariate expression on how deviant the chemical composition of the plant community was from the average across plots and years. An analysis of variance (ANOVA) was performed on *SDist* as described above.

In order to test whether plant diversity effects on stoichiometry could be explained by mixing plant species only, we compared expected to observed ratios. For the expected ratios, the biomass contribution of each species on the large plots from 2003 to 2006 [23] was multiplied by their element concentrations in monoculture, which were obtained by averaging species replicates from small monoculture plots. Because biomass of the small plots was only analyzed for C and N concentrations from 2003–2006, we lack monoculture information on P-content and thus, the calculation was only done for the C:N ratio in those years. The predicted ratios were plotted against the observed ratios of the years 2003–2006 for plant monocultures and diversity levels of 2, 4, 8 and 16 species mixtures. In addition to perform an ordinary least square regression (OLS), we are assessing the performance of the predicted nutrient ratios by an orthogonal regression (also called ‘total least squares’). In OLS, the resulting coefficient is not independent from the choice of predictor and regressor. Conversely, orthogonal regression makes no assumptions regarding the source of the error. The orthogonal regression was estimated using function ‘princomp’ in R [28]. Confidence intervals for the parameters were estimated from 1000 bootstrap iterations. Additionally, we compared the coefficient of variation (CV) observed in mixtures with a predicted CV derived from species relative cover in mixtures and their monoculture CN ratios.

## Results

### Effects of Plant Species Richness and Functional Richness on Bivariate Nutrient Ratios

Plant diversity effects on plant stoichiometry started to become significant after three years (from 2005 onwards) for both species and functional group richness (MANOVA, Table 1). The significance of species richness and functional richness in the model partly depended on the sequence of terms (Table 1, Table S1 and S2), but we still found significant influence of functional diversity after accounting for species richness and marginally significant richness effects after accounting for functional group richness.

**Table 1:** MANOVA results on bivariate elemental ratios for the years 2003 to 2007.

	May 2003	May 2004	May 2005	May 2006	May 2007
Block	0.543*	0.638***	0.356.	0.415*	0.636***
	(CN,NP,CP,CK,NK)	(CP,CK)	(CP,CK,NK)	(CP)	(CN,CP,CK,NK,PK)
sown diversity	0.079	0.095	0.152.	0.226*	0.301***
			(PK)	(NP,CP,PK)	(NP,CP,PK)
functional group richness	0.147	0.111	0.197*	0.167.	0.296***
			(CP,NK,PK)	(NP,CP)	(CN,NP)
Legume	0.525***	0.287***	0.578***	0.696***	0.706***
	(CN,NP,CK,NK,PK)	(CN,NP,CK,NK,PK)	(all)	(all)	(CN,NP,CK,NK,PK)
Grass	0.200.	0.320***	0.223*	0.385***	0.366***
	(CN)	(CN,CP,PK)	(CN,CP,CK)	(CN,CP,CK)	(CN,CP,CK)

For each factor, the Pillai Trace value and its significance level are given as well as all ratios for which the factor effect was significant at p<0.05. Significance levels: p<0.001 = ***, p<0.01 = **, p<0.05 = *, p<0.1 = .

**Figure 1:**
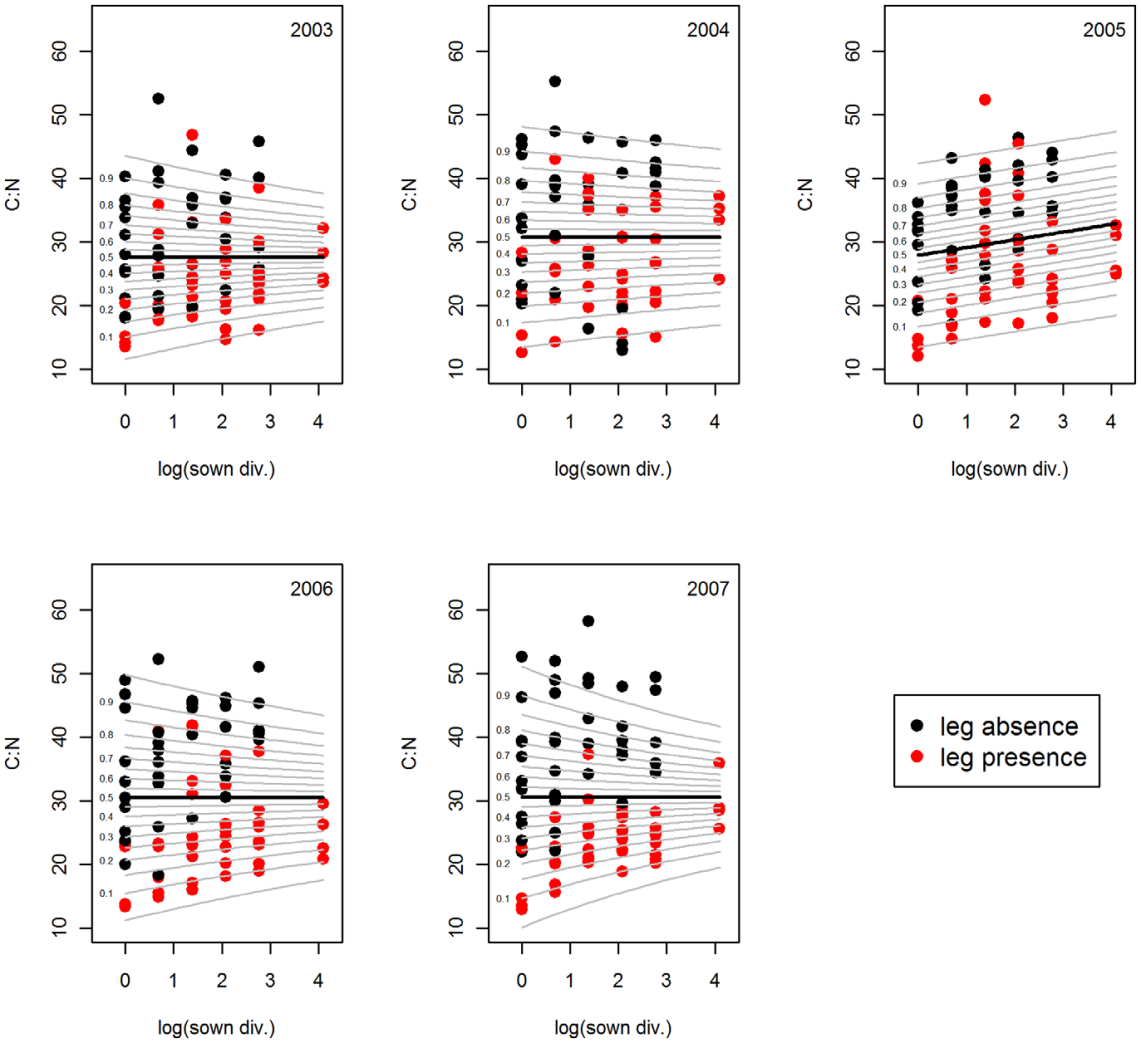
C:N ratio versus plant species richness. GAMLSS (generalized additive model for location scale and shape) model of the molar C:N ratio versus species richness (natural logarithm) of the years 2003–2007. Black line stands for the mean. For better illustration of the variance, percentiles of the standard deviation are given as grey lines. Sown div. = sown diversity, leg = legume.

Plant species richness and functional group richness had similar effects mainly on P-related nutrient ratios, i.e. C:P, N:P and P:K. In 2007, however, the average C:N ratio increased with functional group richness, but not with species richness (Table 1). GAMLSS detected an increasing average C:N with increasing species richness in 2005 only (Fig. 1). In all other years, the variance in C:N ratios declined with increasing plant species richness, whereas the average ratio remained unchanged (Fig. 1).

**Figure 2:**
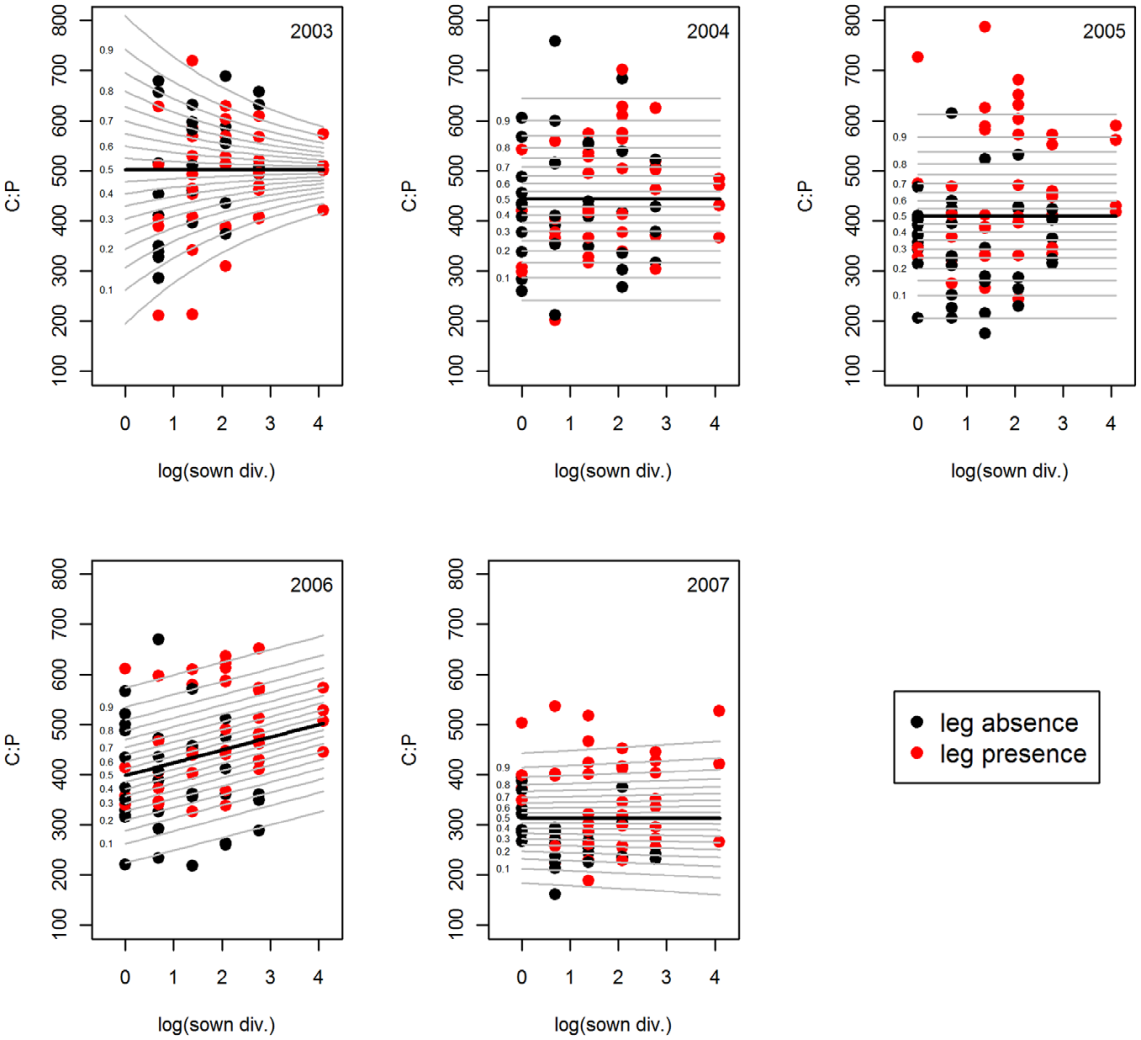
C:P ratio versus plant species richness. GAMLSS (generalized additive model for location scale and shape) model of the molar C:P ratio versus species richness of the years 2003–2007. Black line stands for the mean. For better illustration of the variance, percentiles of the standard deviation are given as grey lines. Sown div. = sown diversity, leg = legume.

The community wide average C:P ratio increased with species richness in 2006 and 2007 (Table 1) and with functional richness in 2005 and 2006. Reversing the order of terms in the model lead to significant increases of C:P with functional group richness from 2005 onwards (see Table S2). GAMLSS detected the same trend only for 2006, when C:P increased by 25% across the richness gradient. Additionally, the C:P ratio became less variable with increasing plant species richness in 2003 (Fig. 2).

Average N:P ratios increased with increasing plant diversity in 2006 and 2007 (both species and functional group richness, Table 1). On average for both years, N:P increased by 26.43% from monocultures to the 60 species-mixture. The same effects were detected in the GAMLSS, which additionally revealed that the variation in N:P ratios decreased with increasing species richness in 2003 and 2005 (see Fig. S1).

**Figure 3:**
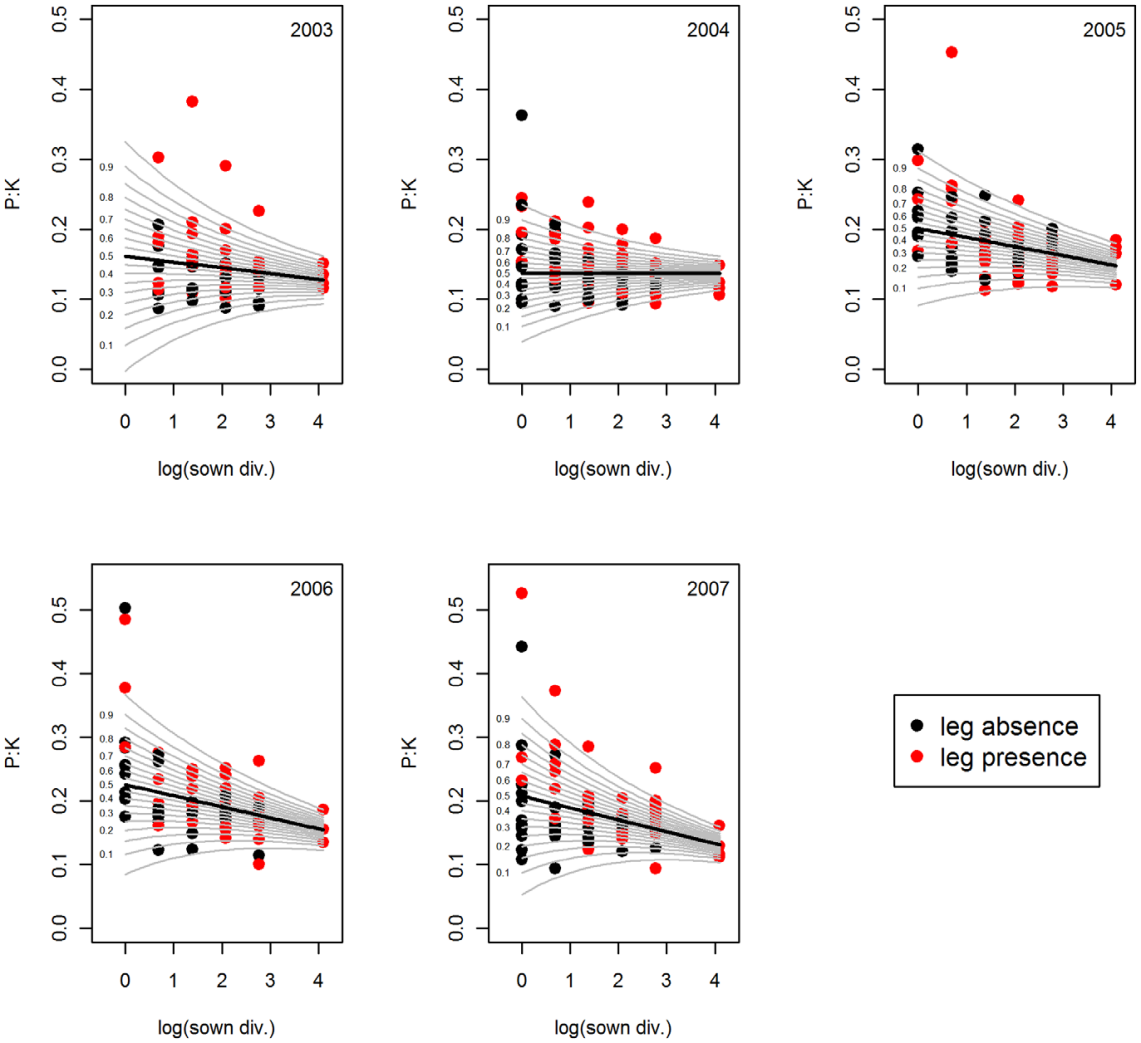
P:K ratio versus plant species richness. GAMLSS (generalized additive model for location scale and shape) model of the molar P:K ratio versus species richness of the years 2003–2007. Black line stands for the mean. For better illustration of the variance, percentiles of the standard deviation are given as grey lines. Sown div. = sown diversity, leg = legume.

The average P:K ratio decreased with increasing plant diversity from 2005 onwards, which was either significant for species richness (Table 1) or functional group richness (see Table S2) depending on term order in the model. The results were consistent in the GAMLSS, which detected a similar negative trend with richness already in 2003. Across the richness gradient, P:K declined by 37.4%. Additionally, the P:K ratios became less variable with increasing richness across all years (Fig. 3).

This decreasing variance with increasing plant diversity was also consistent for the other nutrient ratios containing K (GAMLSS on C:K and N:K, see Figs. S2 and S3), however, these ratios were only marginally shifted by diversity (GAMLSS, C:K, 2006).

**Figure 4:**
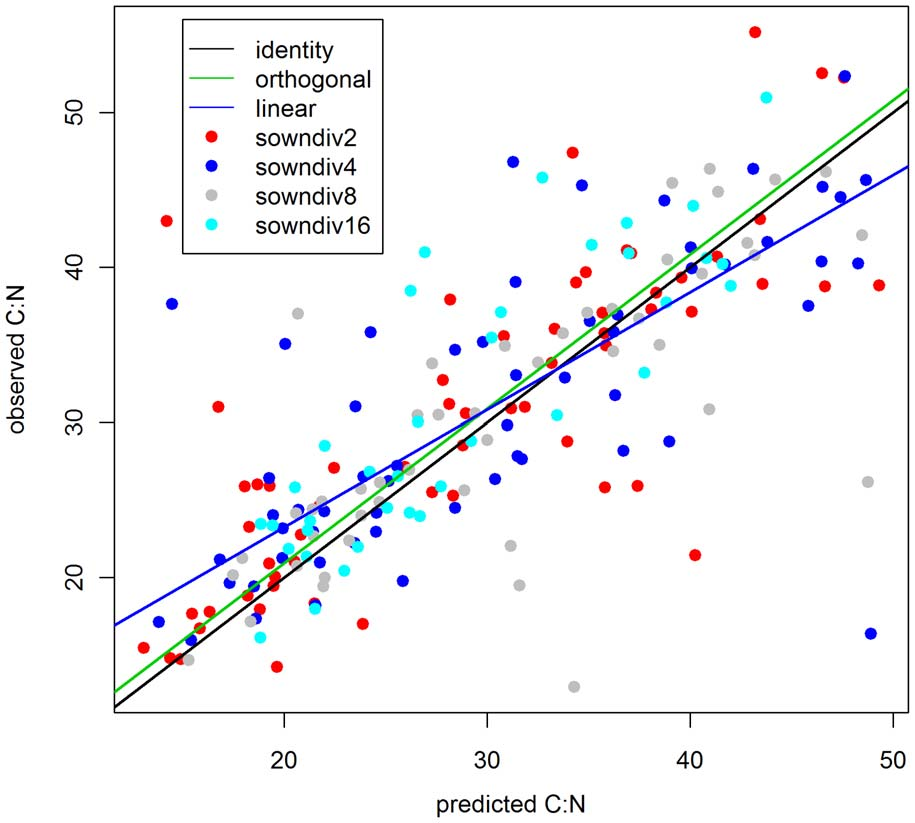
Observed C:N versus predicted C:N ratios. For every measured C:N ratio (2, 4, 8 and 16 species mixtures) the corresponding calculated C:N ratio is shown across years (2003–2006). The green line gives the fit of an orthogonal regression (intercept 1.03 (SD 0.423); estimated C:N 0.999 (SD 0.043). The blue line gives a linear regression (Intercept 8.1448 (SD 1.434); estimated C:N 0.756 (SD 0.046)). For comparison, identity is given by a black line.

**Figure 5:**
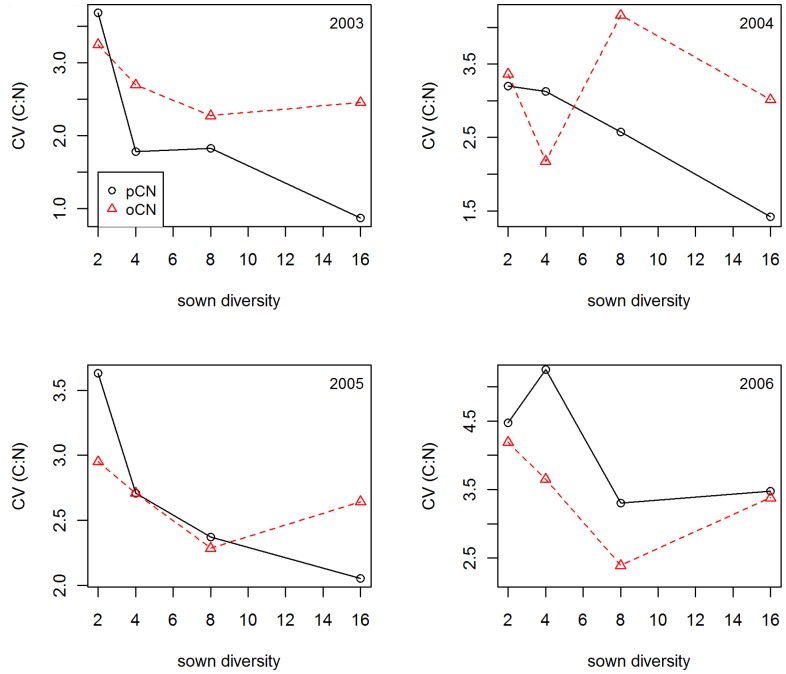
Comparison of the coefficient of variance of observed C:N and predicted C:N ratios. The Coefficient of Variation (CV) of measured (oCN) and calculated C:N (pCN) ratios is shown for different diversity levels (2, 4, 8, and 16 species mixtures) separated by years (2003–2006).

Only for C:N were we able to compare the observed community wide ratios to predicted ratios derived from monocultures (see Fig. 4 and 5). The correlation between observed and predicted ratios was significant (linear regression: intercept 8.1448 (SD 1.434), slope 0.756 (SD 0.046)). The slope of the orthogonal regression is not significantly different from 1. As C:N ratios did not show strong richness effects, an analysis of P-related ratios (C:P, N:P, P:K) would have been more informative, but monoculture P-concentrations were not available.

However, we were able to show that the observed coefficient of variation of C:N was not simply reflecting the relative abundances of species, which was used to estimate predicted CV (Fig. 5). Instead, observed CV tended to be higher than predicted CV in the first years and lower in later years. The observed pattern of CV to species richness also differed from the predicted pattern in the different years.

### Effect of Legumes and Grasses on Bivariate Nutrient Ratios

Legume presence showed highly significant effects on plant chemical composition of almost all elemental ratios in all years. Grass presence had somewhat weaker effects mainly affecting C-related ratios (Table 1). Legume presence significantly reduced C:N by 27.7% across years (Fig. 1), whereas grass presence increased C:N ratios on average by 32.7%. C:P ratios increased by 26.7% in the presence of legumes in 2005 and 2006 (Fig. 2), and by 21.3% in the presence of grasses from 2004 onwards. Community-wide N:P ratios increased with legume presence in all years (Fig. S1), showing increasing effect sizes over time (Table 1), with 28.1% increase in 2003 and 90.1% increase in 2007. Grass presence had no effect on N:P ratios. Legume presence increased P:K (Fig. 3) and N:K ratios (Fig. S3) across all years by 7.4% and 80.4% respectively, whereas grass presence decreased C:K on average by 8.1% from 2005–2007.

### Shifts in Multivariate Nutrient Ratios

**Figure 6:**
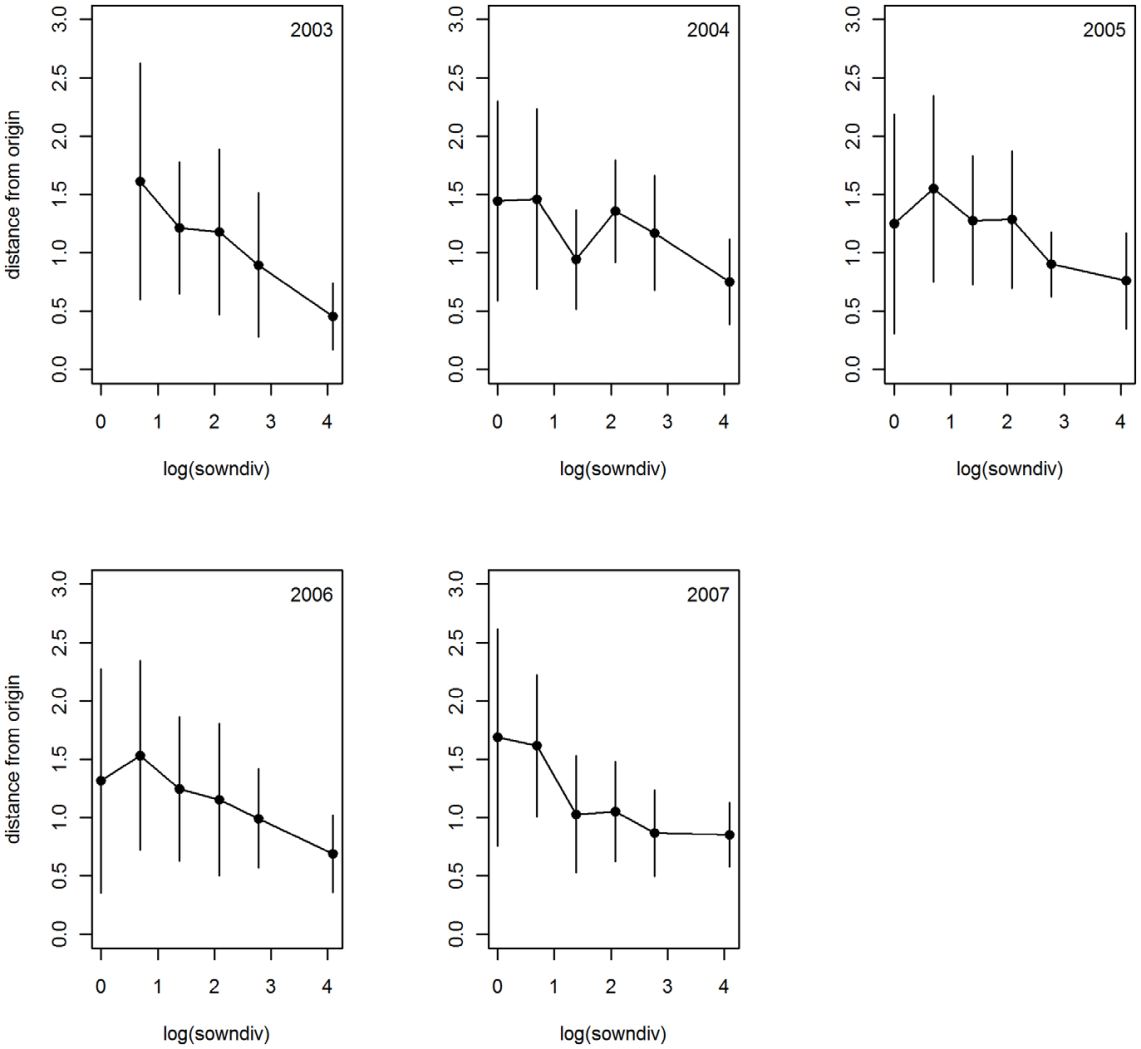
Multielemental stoichiometric distance (SD*ist*) from the origin of the PCA versus sown diversity (log transformed). Error bars are ±1 SD. Axis 1 reflects plant C concentration and opposingly P and K concentration, whereas Axis 2 reflects N alone. The distance from the origin of the PCA (0,0) was used as a measure of stoichiometric imbalance (see methods).

More species-rich assemblages cluster around the origin of the PCA representing all four elements and the species-poor assemblages are more distant from the origin (see Fig. S4). The stoichiometric distance from the origin (*SDist*), which is a measure of stoichiometric imbalance, strongly decreased with increasing plant species richness in all years (Fig. 6). In contrast to the analyses on bivariate ratios, the analysis of *SDist* revealed a significant primary effect of plant diversity throughout time, whereas neither block nor grass presence exerted significant influence (see Table S4). On average, *SDist* decreased by 50% from monocultures to the 60 species-mixture. Legume presence increased *SDist* in the first three years of the experiment, but the effect disappeared thereafter. Functional group richness had the same negative effect on *SDist* as species richness, but only in 2004 and 2006. Thus, increasing species richness alone led to less variable multivariate elemental composition of plants.

## Discussion

Community-wide plant stoichiometry varied with plant diversity (supporting hypothesis H1). We found significant increases in C:P and N:P with either functional group or species richness from 2005 onwards (supporting H1a). When diversity effects on stoichiometry were present, they increased in strength over time (supporting hypothesis H2, Table 1). In addition to shifting mean ratios, plant diversity also reduced the variance in chemical composition (supporting hypothesis H3), which was seen in both multivariate and bivariate analyses of elemental composition. Plant stoichiometry also strongly responded to the identity of functional groups present, confirming that functional composition of plant communities links to chemical composition. Plant diversity effects on average ratios and stoichiometric variance remained when controlling for the presence of certain functional groups. Whereas previous analyses revealed significant diversity effects on elemental content in plants [29], [30] and light use efficiency [31], our study here is substantially novel as it takes an explicit stoichiometric approach to multiple elements.

### Effects of Species Richness on Average Ratios

We found significant effects of species richness or functional group richness on average elemental ratios. The ordering of terms in the model constrained whether species or functional richness effects were dominant, indicating that both aspects were exchangeable. Diversity affected mainly ratios involving P such as C:P and P:K but also C:N. Diversity effects on average stoichiometry showed a lag phase of 3 years, which has also been shown for other responses within the Jena Experiment, e.g. for plant diversity effects on soil microorganisms [32] or soil NO_3_-N [33].

Ptacnik et al. [18] suggested that terrestrial plants increase nutrient uptake more efficiently with increasing diversity than their C fixation, because terrestrial plants show little pigment diversity, but high trait variation in nutrient uptake. Therefore, we expected that an increase in species richness reduces C:nutrient ratios in terrestrial plants, consistent with the results of Novotny et al. [34] who observed that plants respond to changes in diversity by decreasing C:P and C:N ratios from monocultures to mixtures. These results rely on data from only one harvest in 2002, the first year of the experiment. In our study, however, we found the opposite trend after a lag phase of three years. Contrary to our expectation, the diversity effects on stoichiometry may rather reflect allocation patterns in the plant than altered resource efficiency. Within the Jena Experiment, increasing diversity increased community biomass, an effect that increased over time [35]. Plant individuals have to grow taller to successfully compete for light, and in fact this has been shown in the Jena Experiment, where individuals of the same plant species increase in height with increasing diversity [36]. Therefore, plants in more diverse communities have to invest more in rigid stem structure (higher stem:leaf ratios [37], [38]), which increases the C-, over proportional to N- and P-concentrations, resulting in the observed increases in C:N and C:P ratios.

The significant correlation between observed C:N ratios and C:N ratios predicted from relative abundances and monoculture C:N indicates that species composition can explain part of community-wide stoichiometric composition. However, this analysis allows little inference with respect to diversity effects, as C:N was not responding to the richness gradient. Thus, we cannot disentangle whether shifts in average ratios or reduced variance were related to mixture effects from species composition or to a more complete resource use as higher richness increases the probability for complementarity effects for multiple elements. Complementarity in elemental specialization would be a special case of the diversity – multifunctionality relationship [5], [8], as the number of species affecting elemental concentrations in plants will increase with increasing numbers of elements considered. As in our analysis of stoichiometry, complementarity effects on biomass often show a considerable time lag, which has been shown in the Jena Experiment (see below) and in Cedar Creek [39]. For Jena, Marquard et al. [35] showed that complementarity effects increased over time and became stronger than selection from 2005 onwards.

Disentangling between statistical averaging and biological complementarity requires the analysis of species-specific elemental concentrations in all mixtures, which have not been analyzed in this experiment or – to the best of our knowledge – any other BDEF experiment. In a new subproject of the Jena Experiment, the trait-based experiment, these analyses will be available from 2011 onwards.

### Effects of Functional Groups on Average Ratios

Our analysis corroborated previous findings on the effects of certain functional groups on average nutrient concentrations. In line with the ability of legumes to fix atmospheric N_2_ and the associated elevated demand of P, Novotny et al. [34] detected – as we did - that legumes are characterized by lower C:N but higher C:P and N:P ratios than non-legumes (see also [40]). Furthermore, P:K ratios increased significantly across years when legumes were present in the communities, which reflects that communities with legumes had lower concentrations in both P and K, with the reduction being more pronounced with K resulting in higher P per unit K. Within the Jena-Experiment Oelmann et al. [41] found that the absolute aboveground P storage and P exploitation increased if legumes were present in a mixture due to an increased biomass production or due to an increased P demand of legumes for N_2_ fixation [42]. Supporting the latter argument, Roscher et al. [43] described a larger requirement of legumes for P, which might reflect higher P requirement for plants fixing N_2_ [44]. However, our data rather suggest that P and K are diluted in the average community tissue by legume presence, which indicates that higher N_2_ fixation leads to increased biomass production at the expense of reduced tissue concentrations in other elements. The presence of grasses increased the C:nutrient ratios (except for C:K which decreased) across years, corroborating previous findings that the presence of grasses decreases community N and P concentrations because of the lower nutrient demand of monocots compared to dicots [42], [45]. Comparing monocultures of grasses and non grasses within the Jena Experiment, we observed the same pattern as described above but with lower N concentrations.

### Effects of Species Diversity on Stoichiometric Variation

Plant diversity was the most important factor constraining stoichiometric variance. There was no effect of grass presence on stoichiometric variance, whereas legume presence had either no or positive effects on variability, i.e. nutrient ratios tended to be less confined with legumes.

We cannot rule out that the diversity effect on variance is based on an averaging effect of species-specific stoichiometry, which would be a statistical effect without biological mechanism. Comparing observed CV of community C:N and predicted CV based on relative abundance and monoculture C:N, we find little evidence that the diversity effect on C:N variance is a statistical averaging effect alone. Especially after a time-lag, observed CV tended to be consistently smaller than predicted CV, indicating that the common resource use of mixtures is less variable than predicted by species composition alone. This might indicate complementarity effects of resource use as discussed above.

### Consequences of Diversity-related Changes in Stoichiometry

Irrespective whether plant diversity effects on average stoichiometry and its variance depend on statistical averaging or complementarity, our results indicate that a reduction in plant diversity leads to shifting nutrient ratios and increasing variance in elemental composition of plants. Therefore, we can expect subsequent changes on consumer levels, especially for generalist consumers, as herbivores respond strongly to the elemental concentrations of their food by altering individual consumption and area-specific grazing [10]. Extrapolating from ecosystem comparisons [13], higher C:P ratios in the biomass will result in decreased importance of herbivory but increased importance of decomposition and detritivory.

The enhanced productivity paralleled by elevated C:P ratios allows making different predictions how the loss of plant species might affect secondary production in grasslands, which might increase with increasing food quantity but decreases with decreasing food quality. The net effects of these contrasting changes have to be analyzed. Furthermore, we predict that herbivore body size will mediate the effects of increased variance in plant elemental composition in species-poor grasslands. Large, long-lived herbivores will potentially integrate across the increased stoichiometric variability in low diversity plant assemblages, but for small, short-lived herbivores these assemblages could represent a less reliable food environment with patches differing in food quality.

## Supporting Information

Figure S1 **N:P ratio versus plant species richness.** GAMLSS (generalized additive model for location scale and shape) model of the molar N:P ratio versus species richness of the years 2003–2007. Black line stands for the mean. For better illustration of the variance, percentiles of the standard deviation are given as grey lines. Sown div. = sown diversity, leg = legume.
(TIF)

Figure S2 **C:K ratio versus plant species richness.** GAMLSS (generalized additive model for location scale and shape) model of the molar C:K ratio versus species richness of the years 2003–2007. Black line stands for the mean. For better illustration of the variance, percentiles of the standard deviation are given as grey lines. Sown div. = sown diversity, leg = legume.
(TIF)

Figure S3 **N:K ratio versus plant species richness.** GAMLSS (generalized additive model for location scale and shape) model of the molar N:K ratio versus species richness of the years 2003–2007. Black line stands for the mean. For better illustration of the variance, percentiles of the standard deviation are given as grey lines. Sown div. = sown diversity, leg = legume.
(TIF)

Figure S4 **Factor analysis (PCA) of multiple elements across years.** For factor loadings see Table S3.
(TIF)

Table S1 MANOVA results on bivariate elemental ratios excluding 60 species mixtures. For each factor, the Pillai Trace value and its significance level are given as well as all ratios for which the factor effect was significant at p<0.05. Significance levels: p<0.001 = ***, p<0.01 = **, p<0.05 = *, p<0.1 = .
(DOCX)

Table S2 MANOVA results on bivariate elemental ratios with changed order of effects. For each factor, the Pillai Trace value and its significance level are given as well as all ratios for which the factor effect was significant at p<0.05. Significance levels: p<0.001 = ***, p<0.01 = **, p<0.05 = *, p<0.1 = .
(DOCX)

Table S3 Factor loadings for PCA analysis.
(DOCX)

Table S4 ANOVA F-values calculating stoichiometric deviance as distance from CA origin.
(DOCX)
