# Supplementary figures and images for: Biodiversity Effects on Plant Stoichiometry
Source: PLoS One. 2013 Mar 4;8(3):e58179. doi: 10.1371/journal.pone.0058179 (PMC3587429; doi:10.1371/journal.pone.0058179)

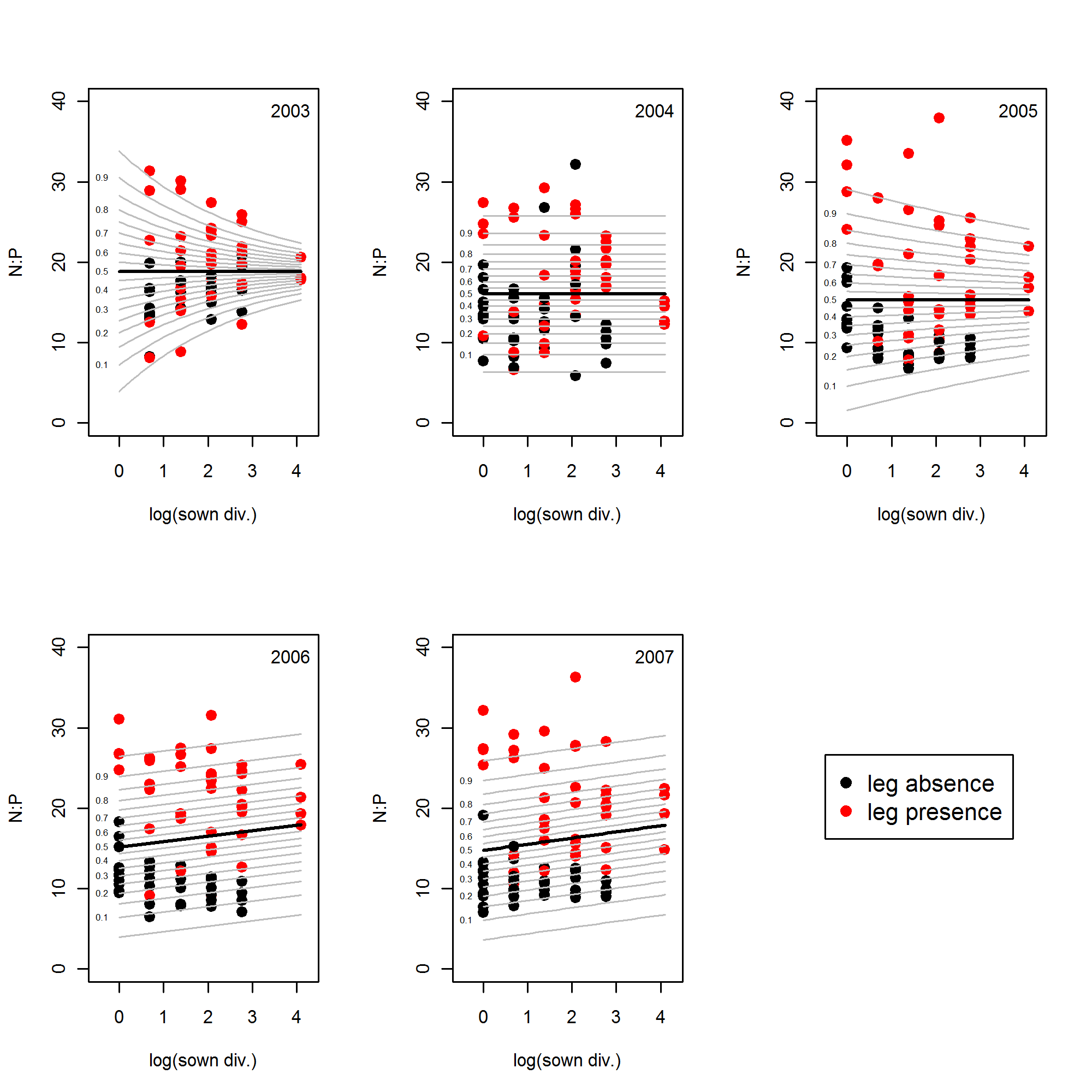

Supplement: Figure S1 — N:P ratio versus plant species richness. GAMLSS (generalized additive model for location scale and shape) model of the molar N:P ratio versus species richness of the years 2003–2007. Black line stands for the mean. For better illustration of the variance, percentiles of the standard deviation are given as grey lines. Sown div. = sown diversity, leg = legume. (TIF) [file pone.0058179.s001.tif]

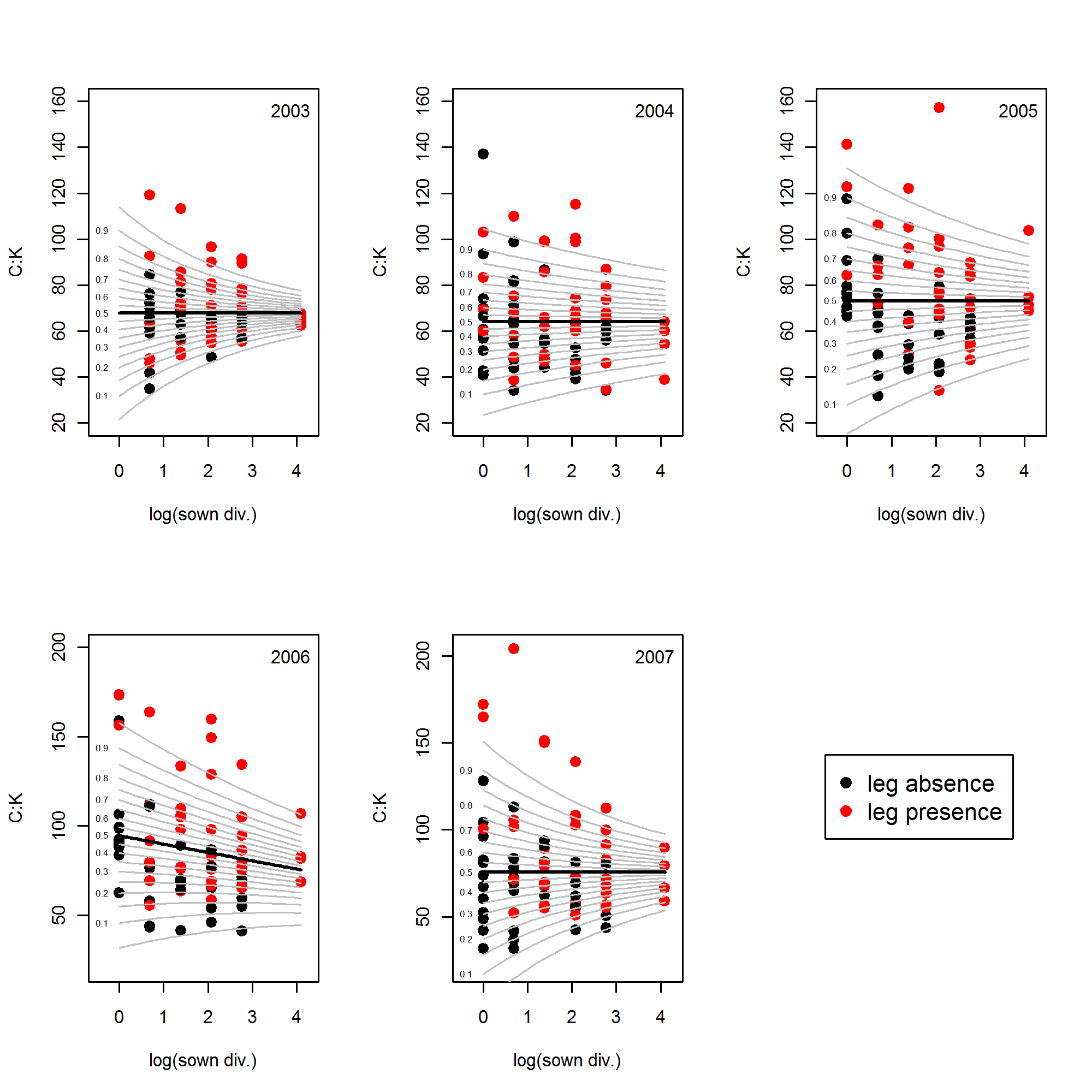

Supplement: Figure S2 — C:K ratio versus plant species richness. GAMLSS (generalized additive model for location scale and shape) model of the molar C:K ratio versus species richness of the years 2003–2007. Black line stands for the mean. For better illustration of the variance, percentiles of the standard deviation are given as grey lines. Sown div. = sown diversity, leg = legume. (TIF) [file pone.0058179.s002.tif]

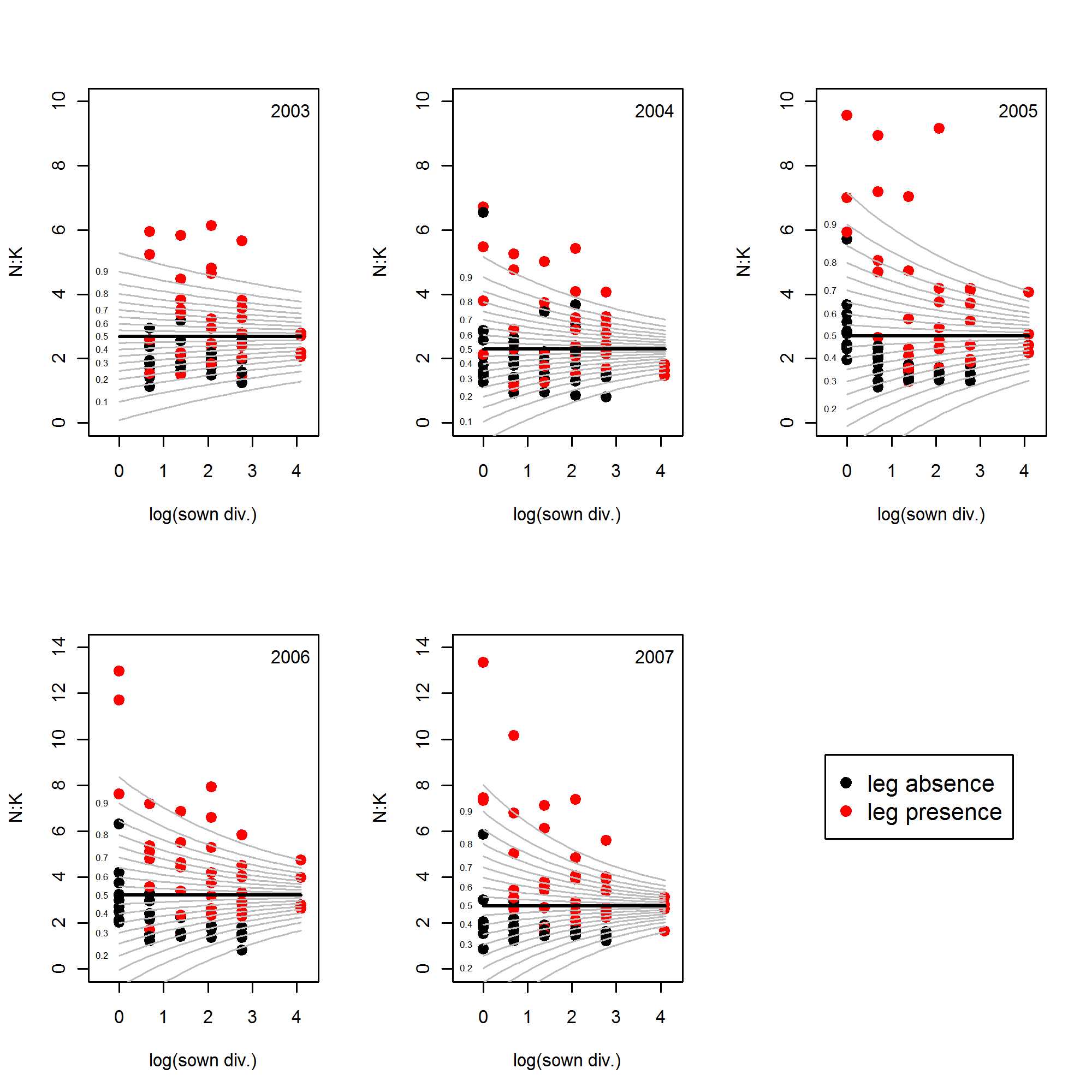

Supplement: Figure S3 — N:K ratio versus plant species richness. GAMLSS (generalized additive model for location scale and shape) model of the molar N:K ratio versus species richness of the years 2003–2007. Black line stands for the mean. For better illustration of the variance, percentiles of the standard deviation are given as grey lines. Sown div. = sown diversity, leg = legume. (TIF) [file pone.0058179.s003.tif]

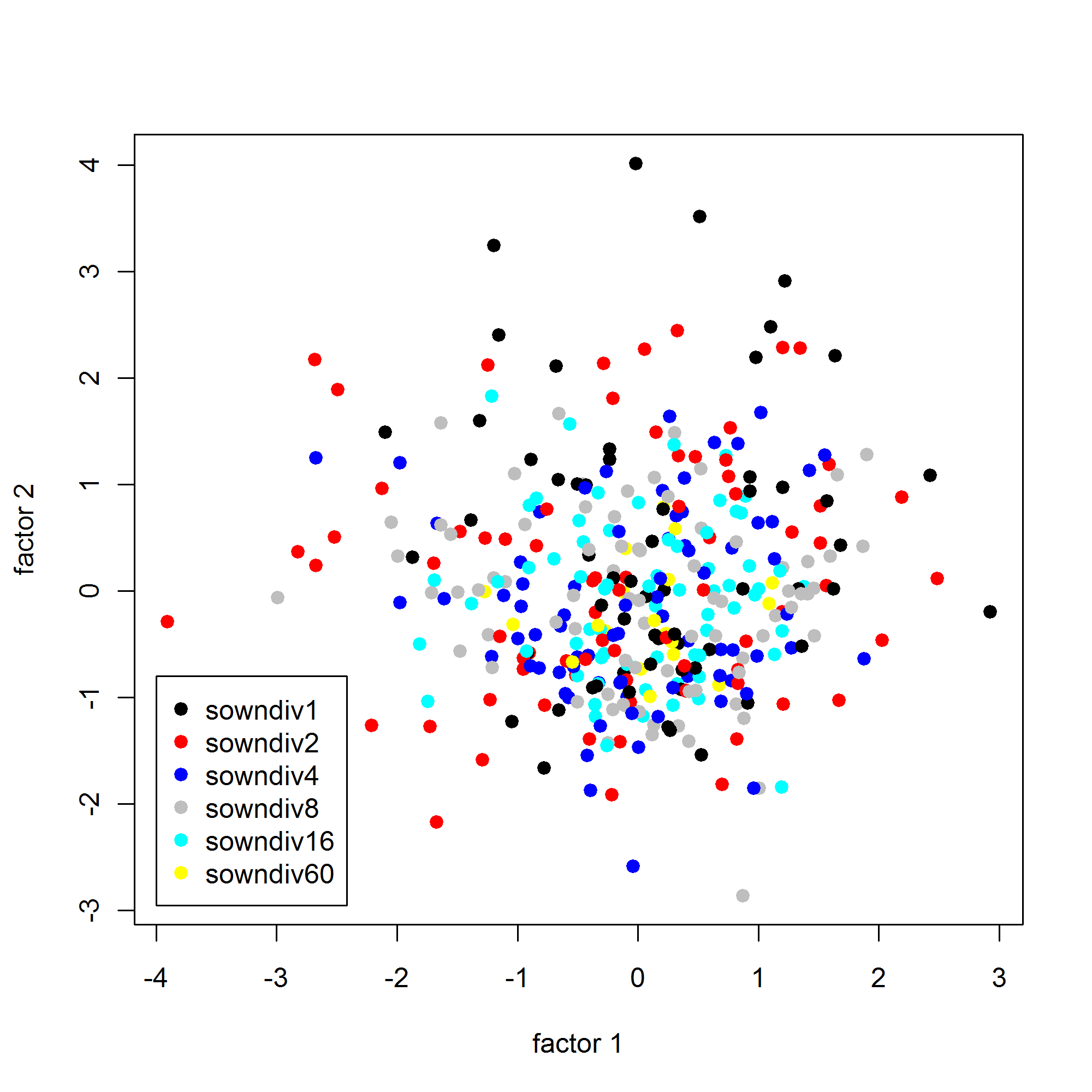

Supplement: Figure S4 — Factor analysis (PCA) of multiple elements across years. For factor loadings see Table S3. (TIF) [file pone.0058179.s004.tif]
