# Supplementary material for: Biodiversity Effects on Plant Stoichiometry
Source: PLoS One. 2013 Mar 4;8(3):e58179. doi: 10.1371/journal.pone.0058179 (PMC3587429; doi:10.1371/journal.pone.0058179)
Supplement: Table S1 — MANOVA results on bivariate elemental ratios excluding 60 species mixtures. For each factor, the Pillai Trace value and its significance level are given as well as all ratios for which the factor effect was significant at p<0.05. Significance levels: p<0.001 = ***, p<0.01 = **, p<0.05 = *, p<0.1 = . (DOCX) [file pone.0058179.s005.docx]

**Table S1**

|  | may 2003 | may 2004 | may 2005 | may 2006 | may 2007 |
| --- | --- | --- | --- | --- | --- |
| block | 0.570* | 0.665*** | 0.363 | 0.437* | 0.651*** |
|  | (CN,NP,CP,CK,NK) | (CP,CK) |  | (CN,CP) | (CN,CP,CK,NK,PK) |
| sown diversity | 0.118 | 0.139 | 0.207* | 0.206* | 0.138 |
|  |  |  | (CK,NK,PK) | (CK,NK,PK) |  |
| functional group richness | 0.151 | 0.094 | 0.169. | 0.158 | 0.303*** |
|  |  |  | (CP,PK) |  | (CN,NP) |
| legume | 0.539*** | 0.287** | 0.582*** | 0.707*** | 0.711*** |
|  | (CN,NP,CK,NK,PK) | (CN,NP,CK,NK,PK) | (all) | (all) | (CN,NP,CK,NK,PK) |
| grass | 0.213. | 0.329*** | 0.219* | 0.389*** | 0.369*** |
|  | (CN) | (CN,CP,PK) | (CN,CP,CK) | (CN,CP,CK) | CN CP CK NK PK |
