# Supplementary material for: Biodiversity Effects on Plant Stoichiometry
Source: PLoS One. 2013 Mar 4;8(3):e58179. doi: 10.1371/journal.pone.0058179 (PMC3587429; doi:10.1371/journal.pone.0058179)
Supplement: Table S2 — MANOVA results on bivariate elemental ratios with changed order of effects. For each factor, the Pillai Trace value and its significance level are given as well as all ratios for which the factor effect was significant at p<0.05. Significance levels: p<0.001 = ***, p<0.01 = **, p<0.05 = *, p<0.1 = . (DOCX) [file pone.0058179.s006.docx]

**Table S2**

|  | may 2003 | may 2004 | may 2005 | may 2006 | may 2007 |
| --- | --- | --- | --- | --- | --- |
| block | 0.543* | 0.638*** | 0.356. | 0.415* | 0.636*** |
|  | (CN ,NP,CP,CK,NK) | (CP,CK) | (CP,CK,NK) | (CP) | (CN,CP,CK,NK,PK) |
| functional group richness | 0.154 | 0.144 | 0.278** | 0.297*** | 0.403*** |
|  |  | (PK) | (CP,NK,PK) | (NP,CP,PK) | (CN,NP,CP,PK) |
| sown diversity | 0.071 | 0.058 | 0.037 | 0.064 | 0.151. |
|  |  |  |  |  |  |
| legume | 0.525*** | 0.287*** | 0.578*** | 0.696*** | 0.706*** |
|  | (CN,NP,CK,NK,PK) | (CN,NP,CK,NK,PK) | (all) | (all) | (CN,NP,CK,NK,PK) |
| grass | 0.200. | 0.320*** | 0.223* | 0.385*** | 0.366*** |
|  | (CN ) | (CN,CP,PK) | (CN,CP,CK) | (CN,CP,CK) | (CN,CP,CK) |
