# Supplementary material for: Biodiversity Effects on Plant Stoichiometry
Source: PLoS One. 2013 Mar 4;8(3):e58179. doi: 10.1371/journal.pone.0058179 (PMC3587429; doi:10.1371/journal.pone.0058179)
Supplement: Table S3 — Factor loadings for PCA analysis. (DOCX) [file pone.0058179.s007.docx]

**Table S3**

|  |  |  |  |  |  |  |  |  |  |  |
| --- | --- | --- | --- | --- | --- | --- | --- | --- | --- | --- |
|  | may 2003 | | may 2004 | | may 2005 | | may 2006 | | may 2007 | |
|  | factor 1 | factor 2 | factor 1 | factor 2 | factor 1 | factor 2 | factor 1 | factor 2 | factor 1 | factor 2 |
| C | 0.889 | 0.047 | 0.534 | 0.591 | 0.785 | -0.168 | 0.783 | -0.073 | -0.782 | 0.074 |
| N | 0.245 | -0.852 | -0.092 | 0.829 | 0.306 | 0.940 | 0.443 | 0.875 | -0.341 | 0.890 |
| P | -0.412 | -0.770 | -0.779 | 0.417 | -0.820 | 0.298 | -0.800 | 0.456 | 0.718 | 0.546 |
| K | -0.884 | 0.170 | -0.892 | -0.096 | -0.897 | -0.099 | -0.844 | -0.040 | 0.897 | -0.034 |
| eigenvalue | 1.802 | 1.349 | 1.697 | 1.221 | 2.187 | 1.010 | 2.162 | 0.981 | 2.049 | 1.097 |
| % explained variance | 0.450 | 0.337 | 0.424 | 0.305 | 0.547 | 0.253 | 0.540 | 0.245 | 0.512 | 0.274 |
|  |  |  |  |  |  |  |  |  |  |  |
