# Supplementary material for: Biodiversity Effects on Plant Stoichiometry
Source: PLoS One. 2013 Mar 4;8(3):e58179. doi: 10.1371/journal.pone.0058179 (PMC3587429; doi:10.1371/journal.pone.0058179)
Supplement: Table S4 — ANOVA F-values calculating stoichiometric deviance as distance from CA origin. (DOCX) [file pone.0058179.s008.docx]

**Table S4**

| year | block | log (sown diversity) | functional group richness | legume | grass |
| --- | --- | --- | --- | --- | --- |
| 2003 |  | 11.05 ** |  | 5.00 * |  |
| 2004 |  | 3.90 + | ß 3.78 + | 4.78 * |  |
| 2005 |  | 4.99 * |  | 5.23 * |  |
| 2006 | 2.39 + | 5.51 * | ß 3.47 + |  |  |
| 2007 |  | 20.39 *** |  |  |  |
